# Magendie’s Introductory Lecture at the College of France

**Published:** 1846-07

**Authors:** 


### SELECTIONS

FROM

### AMERICAN AND FOREIGN JOURNALS.

---

*Magendie's Introductory Lecture at the College of France.* The following remarks, which we extract from the Epoque, were made by Magendie, on opening his course of physiology at the College de France:

I have long been in the habit previous to resuming my lectures, of devoting a few minutes to an inquiry into the actual state of medicine, and to an examination of the modifications which the theory and the practice of medical science may have undergone during the preceding year. This retrospective analysis will to-day furnish us with a certain number of reflections, the importance of which it would be in vain to endeavor to conceal, referring as they do both to the present and the future state of the profession.

Medicine and medical practitioners have attracted much attention during the last year. Many members of our profession have sat in congress, government has issued committees, the ministers have made solemn promises, and it is said that a law to regulate our destinies will shortly be presented to the Chambers. Everything, therefore, seems to smile upon us, and the wind swells our sails. But in the midst of these favorable circumstances—of these happy omens, alarming symptoms are discerned. The future prospects of medicine are the subject of deliberation, but ought we not sooner to deliberate on its existence.

Medicine can only exist but inasmuch as patients have faith in it, and claim its assistance. It is not by theories that it lives, but by clients. Now it is impossible to conceal to ourselves that, at present, a certaiu proportion of the public abandons classical medicine, ironically called *old medicine*, and throws itself into the arms of new systems, thereby firmly believing that it associates itself to the progress of intellect.

Homœopathy—for it is more especially to it that I allude—aspires to nothing else than to overthrow the entire medical edifice with the arms of ridicule and contempt. And do you know how many specifics it numbers? Three hundred and fifty! With a few globules of aconite, at the dose of a billionth of a grain, it produces the same effects as a bleeding of twelve or sixteen ounces. You exhaust your intellectual energies to find out the seat and the nature of a disease.—Vain efforts! Homœopathy shows that every morbid symptom has its origin in psora, a kind of imponderable agent, which therapeutic substances reduced to unimaginable proportions alone can combat. Thus it is only with infinitely small means that infinitely large effects can be produced.

But, you will say, the persons who believe such absurdities are poor dupes, and the men who prey upon their weakness, hardened quacks. You are perhaps right; but listen to those whom you meet in society, and you will be surprised to hear of the wonderful cures that homœopathy has performed. No doubt its promises are often cruelly falsified, but the failures are but little spoken of. Moreover, we must not deny that many patients have recovered their health, in a most unhoped for manner, whilst under homœopathic treatment. It is easy to understand that enthusiasm or the love of gain should take advantage of such facts to exalt the marvels of homœopathy. This brings us back to a question which I have often raised, and which I have endeavored to elucidate by experiments for the last ten years—viz: what is the influence of treatment on the progress of disease?

In hospitals, as well as in private practice, we must first take into consideration the influence of the mind of the patient. Now there can be no doubt but that a patient who takes a medicine, experiences immediate benefit, from the conviction that it will favorably modify his disease. If this favorable result takes place, what has been the real share of the medicinal substance administered! Medical men are always inclined to attribute the cure of the disease they treat to the means which they have employed; but recollect that disease generally follows its course, without being influenced by the medication employed against it. Thus it is that you are often much deceived. A given medicine will succeed in an apparently serious case, and will fail in another case of a less dangerous character, without your being authorized to attribute to yourself in any way the success or the failure.

These reflections explain at once the cures of which homœopathy is so proud. Homœopathy, instead of bleeding a patient, will place gravely on his tongue a globule of aconite, which he will swallow with confidence and faith. You then see the disease improve. But it would have improved just as well without globules, provided some singular operation had struck the imagination of the patient. It really is too great a stretch of credulity to believe that a globule prepared by the formulæ of Hahnemann can contain any active principle. But, on the other hand, any one who has seen disease, must at once admit that this same globule may exercise, through the imagination, a powerful moral effect. You must not, indeed, accuse me of partiality towards homœopathy, when I state that I firmly believe that a physician would cure a patient sooner with globules, if the patient has faith in them, than with the most appropriate medicinal substances, if he distrusted their action.

What I state respecting medicinal substances is equally applicable to bleeding. A patient is seized with the symptoms to which the term inflammatory has been applied, and asks to be bled, believing that the loss of blood will cure him. You open a vein, and the abstraction of a certain quantity of the vital fluid is followed by an amelioration of the symptoms. But take care how you interpret the fact; the improvement may be owing to the moral effect produced, more than to the venesection. I will mention as a proof what I have often observed in my wards at the Hotel Dieu. A patient laboring under acute disease—pneumonia for instance—enters the hospital, believing firmly that he ought to be bled; I bleed him, but merely to the extent of two or three ounces; too small a quantity for the circulation to be in the least influenced by its abstraction. Nevertheless, the patient becomes more calm, and says he is better. A mere trial of bleeding will thus often suffice to arrest the progress of a disease which under another physician would be treated by abundant depletion. For more than ten years I have not found it necessary to have recourse to copious bleeding; in other words, I have rather endeavored to act on the mind of the patient than on the circulation, and I have no hesitation in asserting that my practice has not been the less successful. Indeed, were I to tell you my mind entirely, I should say that it was more especially in the hospitals, in which the most active treatment is adopted, that the mortality is the most considerable. You must not, however, think that I wish to proscribe bleeding; I only wish to warn you against the deductions that may be drawn from its results.

After mentioning various other pseudo-medical delusions, Magendie continued:

It is with regret I have laid before you this melancholy picture of the deplorable abuses which exist in the medical world; I am afraid no laws on the practice of medicine will be able to eradicate them. The quack will always contrive to elude the wisest regulations, for he knows that patients want to be deceived. Yes, gentlemen, we love error, and often refuse to yield to evidence, even when it is proved to us that our good faith and credulity have been imposed upon. Of this fact I will give you the following proof. A lady, a fervent believer in mesmerism, asked her niece, who was poorly, for a lock of her hair, in order to consult a somnambulist. The niece, wishing to try the credulity of her aunt, gave the hair of her maid instead of her own. A renowned somnambulist was consulted, and at once recognised, by the hair of the maid, all the symptoms presented by the niece, whose sufferings she minutely described, to the great edification of the lady. The latter was then informed of the trick that had been played. You would naturally have thought that she would have recognised the imposture of the somnambulist. Not at all; she preferred concluding that the maid servant had the same disease as her niece, and obliged her to submit to a regular treatment, as if she had been poorly, although at that time in the best possible health.

Thus, many causes contribute to throw uncertainty on the result of medical treatment, either rational or empirical.—Sometimes it is the patient who will be deceived; sometimes it is the quack, who deceives knowingly; sometimes it is the conscientious physician, who, nowithstanding his honesty, allows himself to be led into error by an erroneous interpretation of the means employed.

In the presence of such difficulties, it is less desirable for us to endeavor to lay down some law, than to give to our studies a more serious direction. Instead of demonstrating to pupils theories already framed, and often very badly framed, it would be better to teach them rather to study facts, and to take as their guide experimental observation. We have seen the rise, and, in the course of a few years, the fall, of a system which rested entirely on a single undefined word—inflammation. This system, opposed to all physiological ideas, was erroneously called "physiological medicine;" and yet its author was neither devoid of talent nor of knowledge.— Such, no doubt, will be the destiny of those pretended methods of treatment which are, in general, founded on visionary speculations, have fashion for support, and dupes for followers.

As for us, gentlemen, we shall continue, as we have hitherto done, to study phenomena in the organs in which they are accomplished. Although the part of the physician may not be always as efficacious or as active as we could wish, when disease requires assistance, we may still, by well-judged intervention, assist Nature in overcoming the functional derangements which give rise to disease. We shall more especially, therefore, endeavor correctly to appreciate the physiological action of organs. How, in reality, can we have recourse to rational treatment when we are ignorant of the normal conditions of the economy.

It will be perceived that Magendie has taken advantage of the opportunity furnished him by a cursory sketch of the popular medical delusions of the day, to give utterance to peculiar opinions respecting the influence of medicinal agents in the treatment of disease—opinions which stamp him as nearly a sceptic with reference to their therapeutic powers. The partial scepticism of M. Magendie as regards medicinal agents has been long known to French pathologists, and has contributed not a little, with them, to throw discredit on his physiological experiments. He may be considered the most prominent representative of the French 'expectant' school—a school which now numbers but very few votaries among our neighbors, notwithstanding the opinions to the contrary generally entertained in this country. The great reputation of Magendie among British physicians, which induces most medical travelers when in Paris to hasten to his wards at the Hotel Dieu, of which he is one of the medical officers, will partly account for the erroneous views respecting modern French practice that are entertained by many of our countrymen. Magendie has experimentalized himself into the belief that nature requires but little other than dietetic and hygienic assistance when disease is present. Discarding, consequently, the accumulated wisdom of former ages, he treats his patients, conscientiously, according to these views, and it is generally considered by his Parisian colleagues with but indifferent success.

The error into which Magendie has fallen exemplifies the danger of laying too great stress on the results of mere experiments, sometimes as fallacious sources of knowledge as any. The same may be said of the numerical system when pushed to the extreme, and resorted to without due qualification, as a means of therapeutic research. Both these modes of investigation, although admirable in themselves, when abused, oı used as mathematical formulæ, nearly inevitably lead to that most lamentable of medical errors, scepticism in the power of therapeutics.—*London Lancet.*

